# Thrombosis Development After mRNA COVID-19 Vaccine Administration: A Case Series

**DOI:** 10.7759/cureus.41371

**Published:** 2023-07-04

**Authors:** Sundeep Bekal, George Husari, Marcel Okura, Charity A Huang, Mohammed S Bukari

**Affiliations:** 1 Internal Medicine, Community Hospitalist Physician Group, Fresno, USA; 2 Internal Medicine, University of California, San Francisco Fresno, Fresno, USA; 3 Hematology and Oncology, Harbor-University of California Los Angeles Medical Center, Torrance, USA; 4 Hematology and Oncology, University of California, San Francisco Fresno, Fresno, USA

**Keywords:** vaccine associated complications, thrombosis, covid-19 mrna vaccine, venous thromboembolism (vte), covid-19

## Abstract

The coronavirus disease 2019 (COVID-19) pandemic remains one of the largest global health crises of the last century. Fortunately, COVID-19 vaccines have proven to be one of the most promising options in halting the progression of the pandemic. As more and more people receive COVID-19 vaccines, the medical community has learned a great deal about their efficacy and the occurrence of very rare adverse effects. While the number of thromboembolic events post-adenoviral vaccines has been well-documented in the medical literature, there has been limited information regarding thrombosis development after receiving a messenger RNA (mRNA)-based vaccine. This case series highlights four different patients who received an mRNA-based COVID-19 vaccine and subsequently developed venous thromboembolism. Therefore, we hope that after reviewing this article, physicians will be more aware of thrombosis-related developments following mRNA vaccine administration for COVID-19. Fortunately, with early diagnosis and prompt treatment, patients can still expect full recovery from any vaccine thrombosis-associated complications, and the benefits of receiving an mRNA-based COVID-19 vaccine still outweigh the risks of post-vaccination complications.

## Introduction

Coronavirus disease 2019 (COVID-19), caused by the severe acute respiratory syndrome coronavirus 2 (SARS-CoV-2) virus, is undoubtedly the greatest global health crisis in the last century. The World Health Organization and other multinational organizations have issued a global call to action, resulting in increased funding for pandemic-related initiatives, including the production of personal protective equipment, dedicated COVID-19 therapies, and public education.

The advancements in vaccine manufacturing science have led to the development of multiple vaccines using viral vector and messenger RNA (mRNA) technology by various countries and institutions, significantly impacting the course of the pandemic. Vaccination against COVID-19 has proven to be one of the most effective preventive strategies to date [1].

However, the emergence of vaccines has also brought about severe adverse effects, such as venous thromboembolism (VTE) and vaccine-induced immune thrombotic thrombocytopenia (VITT), which quickly led to vaccine hesitancy and temporary halts in vaccination campaigns in certain countries [2,3].

Interestingly, this phenomenon has only been observed following the administration of adenoviral-vectored DNA vaccines like AstraZeneca's ChAdOx1 nCoV-19 and Janssen's Ad26.COV2.S [4]. A 2022 report from the Vaccine Adverse Event Reporting System (VAERS) surveillance system to the CDC (Center for Disease Control) and FDA (US Food and Drug Administration) estimated an incidence of approximately 3.8 per million vaccine doses (approximately 1 in 263,000 vaccine doses) [5].

Although the current medical literature contains numerous case reports demonstrating these outcomes in patients who received an adenoviral vector-based vaccine, there has been limited information on thromboembolic events following mRNA-based vaccines such as the Pfizer BNT162b2 mRNA COVID-19 vaccine and Moderna COVID-19 mRNA-1273 vaccine [6-7]. See et al described in their report that of 57 cases of thrombocytopenia with thrombosis syndrome (TTS) that were reported to the VAERS between December 2020 and September 2021, only three cases were post mRNA COVID-19 vaccine administration [5].

In this report, we present four unique cases of thromboembolism development in patients who recently received an mRNA-derived COVID-19 vaccine. All four patients were vaccinated in different vaccination centers prior to admission, however, they all presented with their VTE diagnosis in the same hospital during various months in the course of one year.

## Case presentation

Case 1

**Figure 1:**
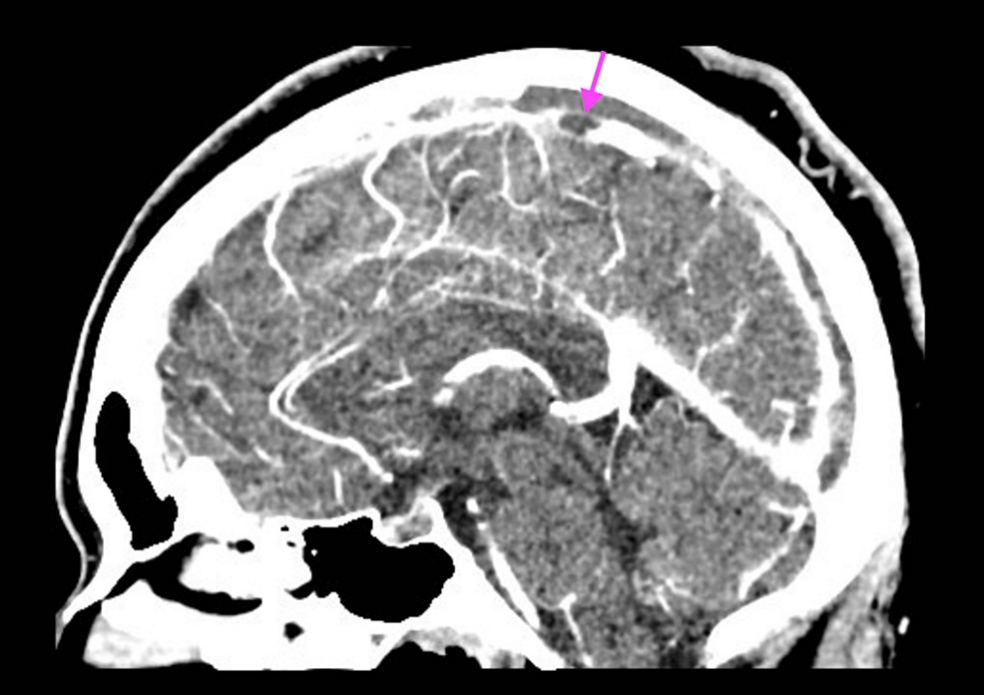
Acute venous dural sinus thrombosis present in the superior sagittal sinus seen on the CT venogram of Case 1

A 31-year-old female with a past medical history of type 2 diabetes mellitus, hyperlipidemia, hypertension, and obesity class II (body mass index of 38) presented with headaches and photophobia two days after receiving the second dose of the Moderna vaccine. She reported no additional provocatory event. Her physical exam is normal. Laboratory workup showed normal complete blood count (CBC) and complete metabolic panel (CMP). Computed tomography (CT) of her head without contrast was obtained, which did not show any acute hemorrhagic or ischemic infarct. A CT venogram of her head was ordered, which revealed acute venous dural sinus thrombosis in the superior sagittal sinus (Figure 1).

**Figure 2:**
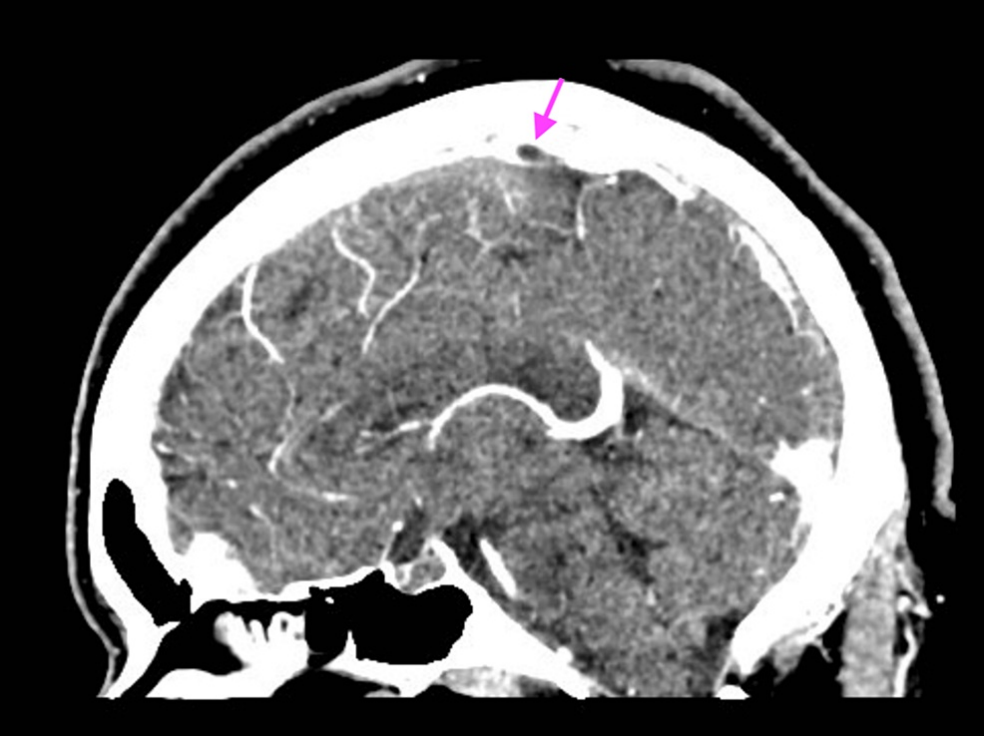
Interval decrease in thrombosis involving the superior sagittal sinus 30 days later in Case 1

Hypercoagulable workup, including immunological evaluation for antiphospholipid syndrome (APS), anti-nuclear antibody (ANA), beta 2 glycoprotein (b2GP), anti-platelet factor 4 (anti-PF4), prothrombin gene mutation, and factor V Leiden, was negative. However, D-dimer was significantly elevated at 1831 ng/mL (Ref: 0-499 ng/mL). She was treated with intravenous argatroban and transitioned to oral apixaban on discharge. A follow-up CT venogram 30 days later showed interval improvement of the clot burden, which correlated with the resolution of her clinical symptoms (Figure 2).

Case 2

**Figure 3:**
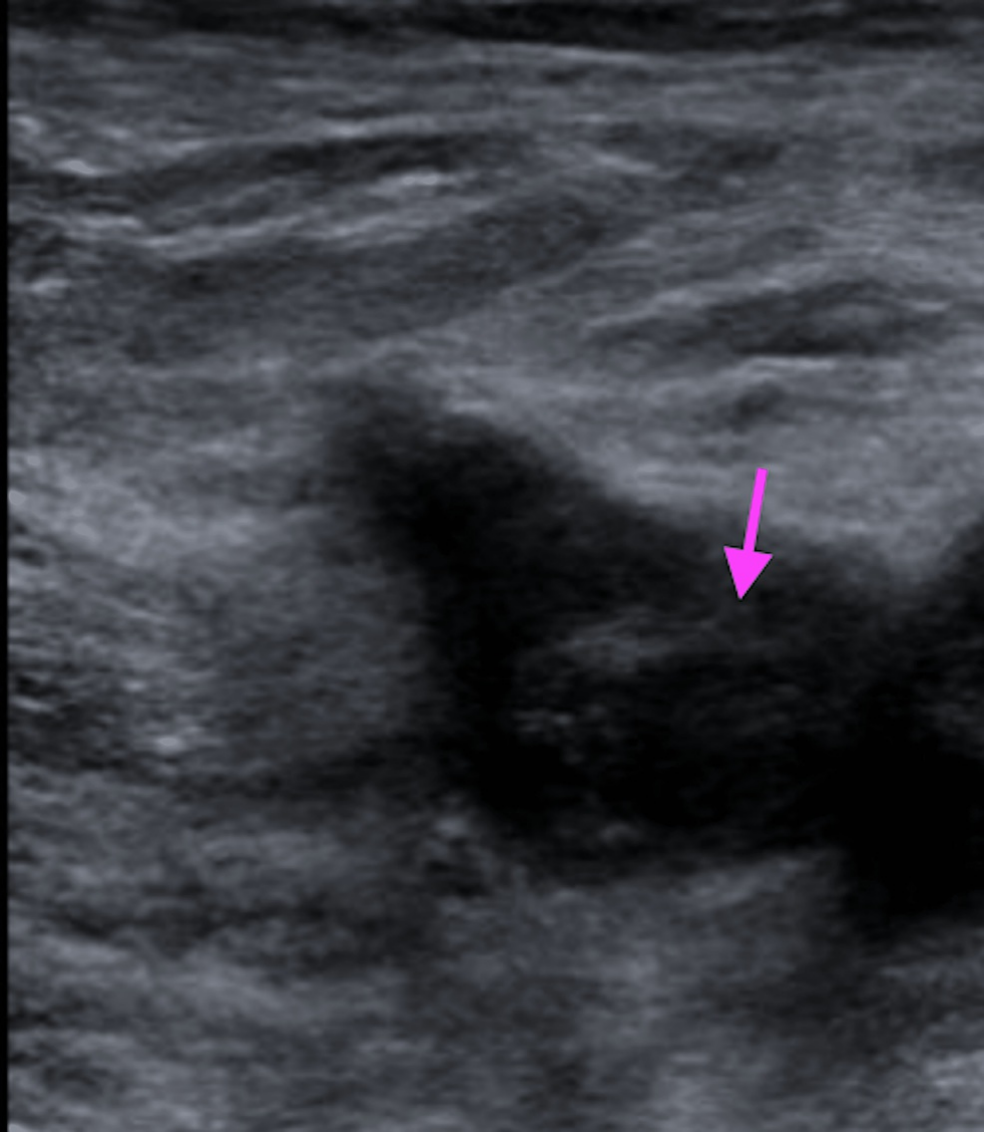
Nonocclusive DVT involving the left common femoral vein seen on ultrasound in Case 2 DVT: deep vein thrombosis

A 75-year-old male with type 2 diabetes mellitus and hypertension presented with a three-day history of left lower extremity swelling that started 10 days after his second dose of the Pfizer vaccine. He reported no other provocatory event. His functional status was noted to be independent and was listed as a level 4 under the Banner Mobility Assessment Tool (BMAT) by physical therapy in the hospital. A physical exam showed diffuse left leg swelling and tenderness. Laboratory evaluations, including CBC, CMP, and a D-dimer, are within normal limits. An ultrasound of his lower extremity illustrated a left common femoral non-occlusive deep vein thrombosis (DVT) (Figure 3).

**Figure 4:**
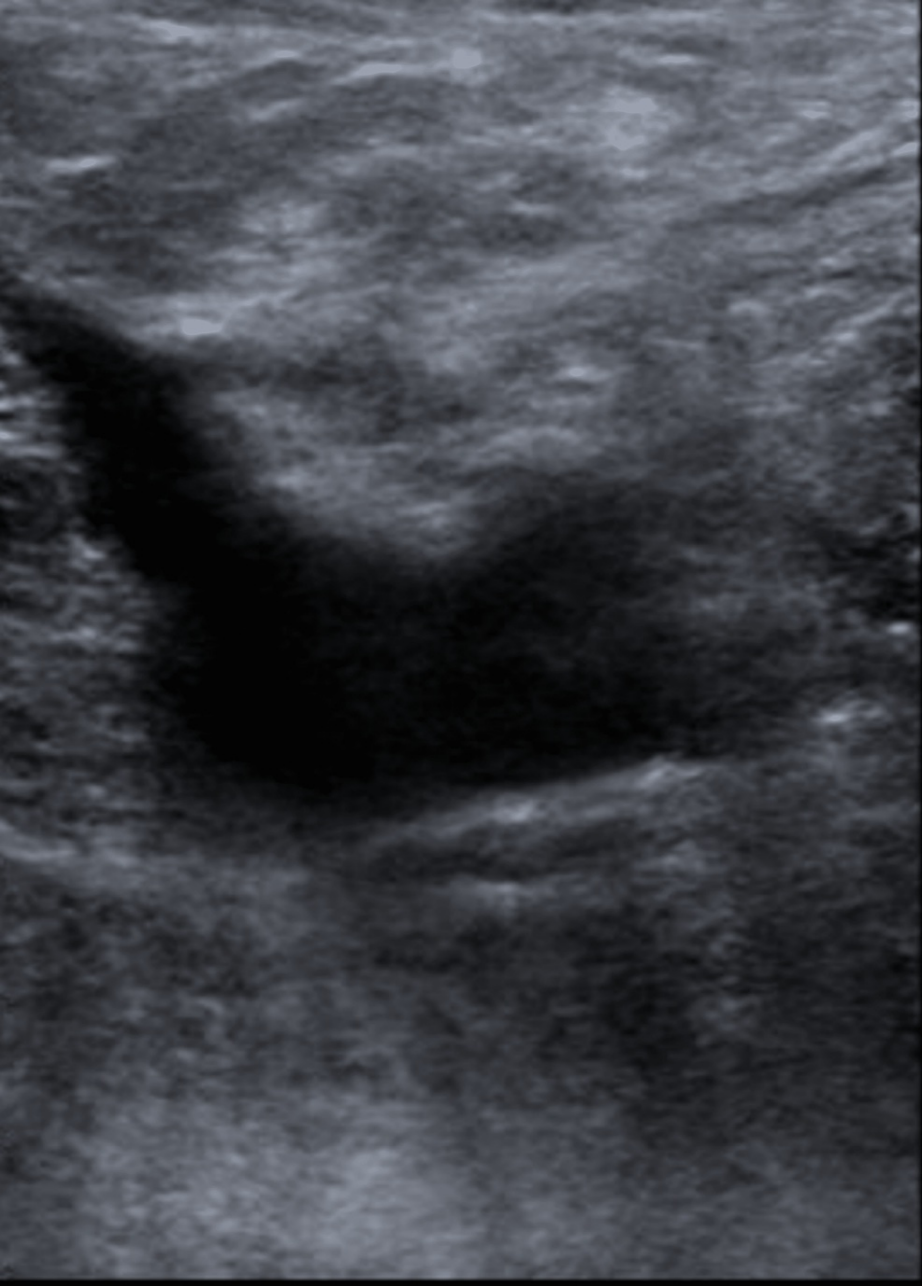
Resolution of thrombosis 90 days later in Case 2

Limited hypercoagulable workup was negative. He received intravenous heparin and was switched to oral apixaban on discharge. A repeat ultrasound was obtained three months later in the clinic, which showed the resolution of the deep vein thrombosis (Figure 4).

Case 3

**Figure 5:**
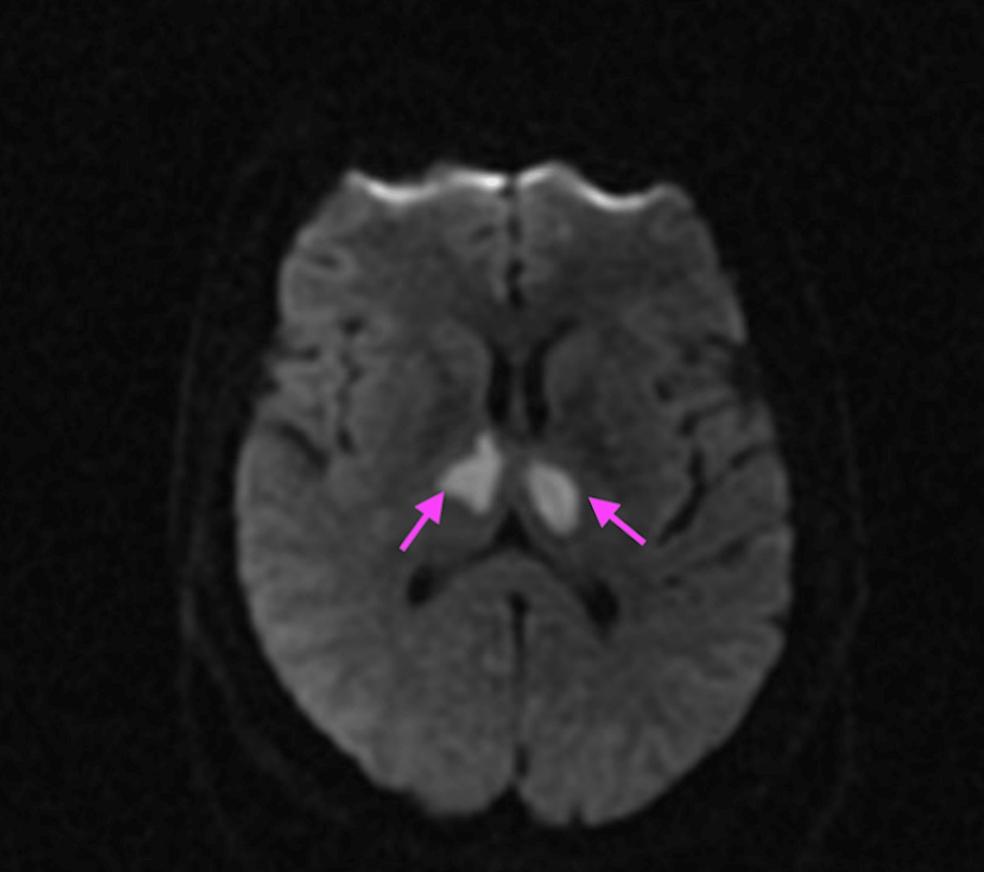
Bilateral thalamic infarcts were seen on magnetic resonance imaging (MRI) in Case 3

A 40-year-old female with no past medical history was found unresponsive two months after her second dose of the Pfizer vaccination. She was intubated for airway protection and admitted to the intensive care unit for a workup of her condition. Laboratory evaluation showed a white blood count of 12.9 x10^3^ μL (Ref: 4.4-11.0 x10^3^ μL) and a normal CMP. Computed tomography (CT) of her brain showed no signs of an acute bleed. A follow-up MRI head was ordered, which commented on bilateral thalamic ischemic infarcts (Figure 5).

**Figure 6:**
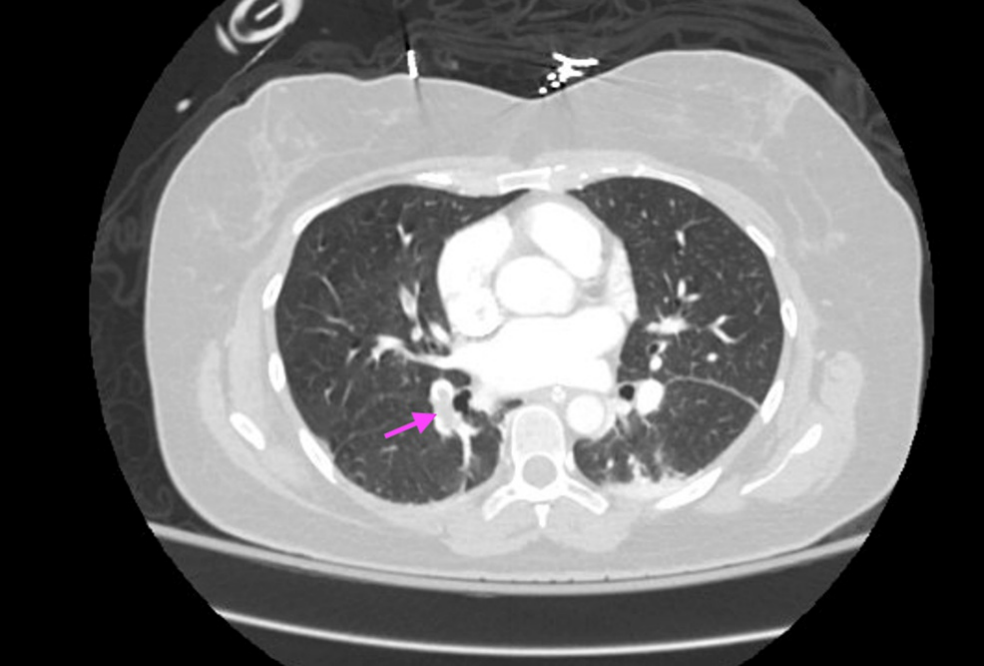
Incompletely occlusive pulmonary emboli involving the right-sided pulmonary arteries seen on CT angiogram of the lungs in Case 3

**Figure 7:**
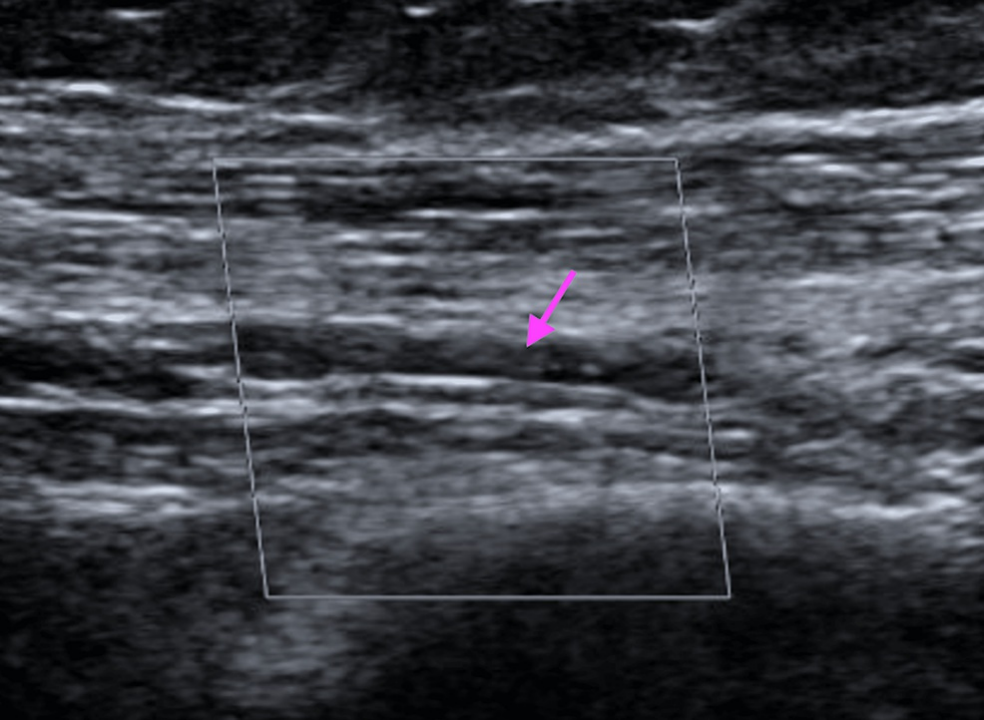
Nonocclusive DVT involving the right mid-brachial vein on ultrasound in Case 3 DVT: deep vein thrombosis

Thromboembolism workup, including an echocardiogram, CT angiogram of the chest, and extremity venous duplex ultrasound showed an intracardiac shunt with patent foramen ovale (PFO), multiple pulmonary emboli in the right lung, and a right brachial vein deep vein thrombosis, respectively (Figures 6, 7).

Extensive hypercoagulable workup including proteins C and S, antithrombin III, lupus anticoagulant panel, prothrombin gene mutation, and factor V Leiden were unremarkable.

She eventually was extubated and was treated with intravenous heparin while in the hospital and later transitioned to oral apixaban upon discharge. Her PFO was closed at three months post-discharge. A follow-up examination five months later showed she had complete resolution of all previously reported DVTs and return to normal neurologic function.

Case 4

**Figure 8:**
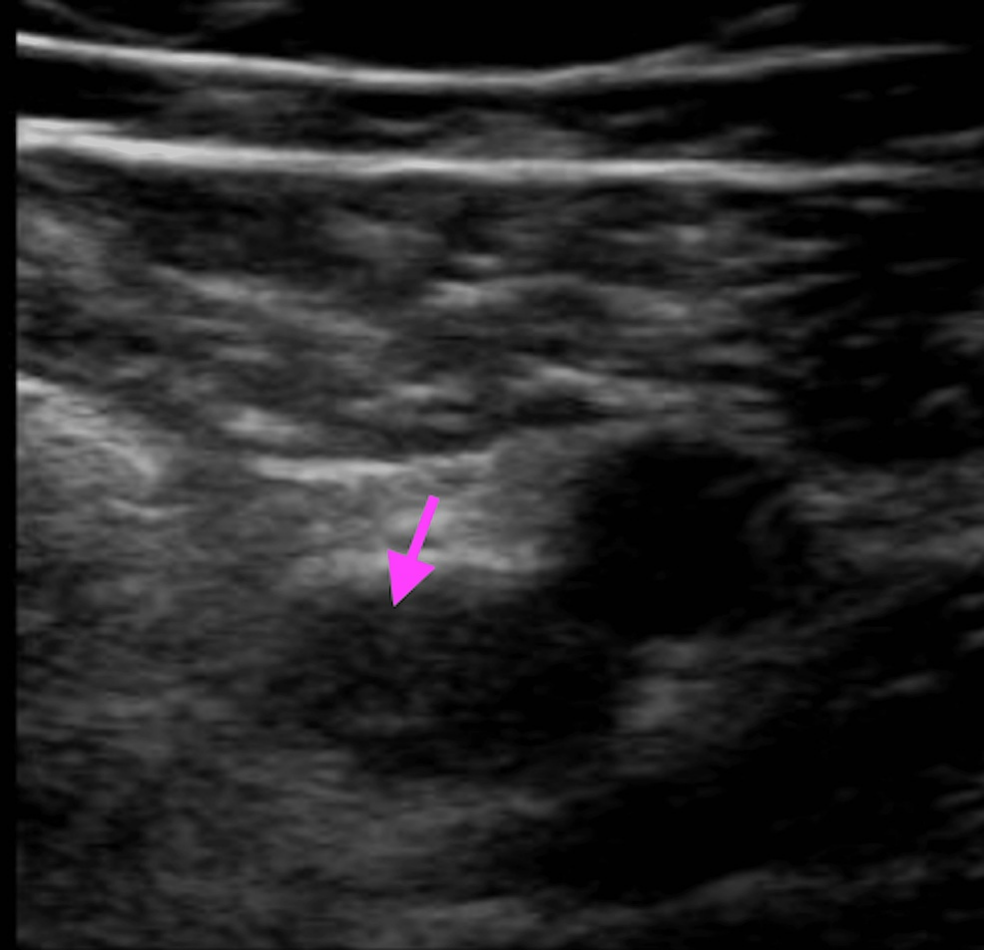
Occlusive deep vein thrombosis involving the right superficial femoral vein on ultrasound in Case 4 DVT: deep vein thrombosis

A 58-year-old male with a history of hyperlipidemia presented to the clinic with right leg swelling seven days after receiving his Moderna vaccine booster. Physical exam was unremarkable except for unilateral right leg swelling and tenderness. Laboratory evaluation, including CBC and CMP, were normal though his D-dimer was elevated at 8233 ng/mL (Ref: 0-499 ng/mL). An ultrasound of his right lower extremity was obtained, which was significant for occlusive deep vein thrombosis involving the middle and lower segments of the right superficial femoral vein (Figure 8).

The patient was promptly started on oral apixaban. A repeat ultrasound was obtained three months later, which showed the resolution of his deep vein thrombosis. A hypercoagulable workup was ordered once he completed three months of apixaban, including protein C, protein S, antithrombin III levels, and a lupus anticoagulant panel; however, these were all within normal limits.

## Discussion

As the number of vaccinations for the COVID-19 virus increases, so does the number of documented side effects. Many different types of adverse effects have been described such as fever, myalgia, headache, weakness, nausea, and anaphylaxis [8]. However, thrombosis-related effects remain one of the major concerns within the community, specifically vaccine-induced immune thrombotic thrombocytopenia (VITT).

Vaccine-induced immune thrombotic thrombocytopenia (VITT), also known as thrombosis with thrombocytopenia syndrome (TTS), is a rare and potentially fatal disorder that was first described in healthy recipients of the COVID-19 vaccine. Two adenoviral vector-based vaccines have been implicated in causing VITT: ChAdOx1 nCoV-19 (AstraZeneca) and Ad26.COV2.S (Johnson & Johnson). The incidence is reported to be around 1 per 100,000 to 250,000 vaccine recipients, although the numbers seem to vary [4]. The syndrome is characterized by severe thrombocytopenia and thrombosis occurring five to 30 days after vaccine administration [9].

There are no well-known risk factors post-vaccination other than female sex and age younger than 60 years [10]. Infections, both localized and systemic, and inflammatory diseases (e.g., rheumatoid arthritis, inflammatory bowel disease) have also been shown to contribute to the development of thrombosis. Other risk factors include cancer, high blood cell counts, and nephrotic syndrome [10].

Although VITT has now become an established entity in the medical community, information regarding thrombosis-related events in the setting of mRNA-derived vaccinations is very limited. Carli et al. reported the first case of an association between venous thromboembolism (VTE) and the Pfizer vaccine in April 2021, describing a 66-year-old female who presented with a right lower extremity DVT 24 hours after the second dose of the vaccine [11]. Andrasaka et al. also reported three cases of VTE in females after Moderna vaccination in January 2022, with the hypercoagulable workup being negative in all three patients [7].

The exact mechanism for mRNA vaccine-associated thrombosis is unclear, and some authors have even proposed that this phenomenon is a separate process entirely from TTS/VITT [12]. Unlike TTS/VITT, which was reported as an adverse reaction after the first dose of the vaccine, our case series illustrates that all thrombosis presentations occurred after the second dose or booster dose.

Studies by Greinacher et al. and Oldenburg et al. have proposed that TTS/VITT is caused by the formation of antibodies against PF4-polyanion complexes as part of the inflammatory reaction and immune stimulation. These antibodies then cause massive platelet activation leading to coagulopathy, similar to the mechanism of heparin-induced thrombocytopenia (HIT) [13-14].

However, the absence of thrombocytopenia in all our cases may suggest a different mechanism of thrombosis. A study by Marshal M. et al. suggested a role of "increased systemic reactogenicity and immunogenicity in younger study participants after mRNA vaccine" [15]. Adverse events occurred more frequently after the second dose and within two days after vaccination such as injection site pain, fatigue, arthralgia, fever, chills, and lymphadenopathy [15]. It is possible that thrombosis development may be an additional rare adverse event related to this theory of systemic reactogenicity. Unfortunately, we cannot infer any causal relation at this time, and more data are required to establish this link.

Thrombosis associated with mRNA-derived vaccines is not yet recognized as a separate entity by formal societies. As a result, no clear guidelines or expert opinions exist on the ideal management. However, what we can infer from our cases is that thromboses should be managed like any other episode of venous thromboembolism. Patients should be started on an anticoagulation agent, and the total duration should be evaluated by the clinician on a case-by-case basis. In all our patients, we observed complete resolution of VTE after three months of therapy, and none of the patients required a longer duration of anticoagulation upon outpatient follow-up after one year. In fact, all four patients returned to their previous level of independent function.

While our case series does note four examples of severe complications secondary to an mRNA-derived vaccine, the case series provides some important information. Early diagnosis combined with a high index of suspicion allowed all patients to be promptly started on therapeutic treatment. In addition, once started on treatment, all four patients displayed no adverse outcomes, and in fact, all had complete recovery when followed up post-hospitalization. Regulatory bodies in the United States and Europe have now concluded that the population benefits of COVID-19 vaccinations outweigh the risk of these rare events [16]. A recent analysis reported that the crude incidence rate of cerebral venous thrombosis hospitalization was 28-32 times higher in patients after a COVID-19 infection compared to patients after receiving an mRNA-based COVID vaccine [17]. Pulmonary embolism (PE) and deep vein thrombosis (DVT) have also been described at a much higher frequency in hospitalized and severe COVID-19 infections compared to those who received an mRNA-based vaccine [18-19]. In addition, the risk of hospitalization or death associated with thromboembolic complications with a COVID-19 infection is potentially higher than those associated with the vaccines [20].

## Conclusions

Despite the increasing number of vaccine administrations, there is still limited information regarding the risk of VTE after receiving an mRNA-based COVID-19 vaccine. As we continue with large-scale vaccinations across the population, it is crucial to report the incidence of post-mRNA COVID-19 vaccine thrombotic events. Despite concerns about VTE development, early diagnosis and prompt treatment can result in favorable outcomes for patients. As such, mRNA-derived vaccines remain our most valuable resource in combating the ongoing pandemic.
